# Looking for methodologies and data pathways used in research to assess the COVID-19 impact: a scientific literature review: Rodrigo Santos

**DOI:** 10.1093/eurpub/ckac129.060

**Published:** 2022-10-25

**Authors:** R Feteira-Santos, Dr Nogueira

**Affiliations:** 1 Área Disciplinar Autónoma de Bioestatística, Instituto de Medicina Preventiva e Saúde Pública, Lisbon, Portugal; 2 Instituto de Saúde Ambiental, Universidade de Lisboa, Lisbon, Portugal

## Abstract

COVID-19 spread worldwide after the surge of a novel coronavirus infecting humans in late 2019, rapidly becoming a pandemic in early 2020. Then, the scientific community started studying SARS-CoV-2 infection to address the uncertainties around the impact of this new disease on the population health and sustain public health measures to mitigate it. As a result, the research on COVID-19 proliferated fast, and the number of related records also multiplied in research databases. By November 2020, one year after starting the outbreak, PubMed counted more than 80 thousand records, which had already tripled by April 2022. Facing that large body of research, the PHIRI WP5 team deployed a literature review to identify research methods and the research paths from the generation and collection of data used to assess the COVID-19 impact on the population's health and well-being. Consequently, this work aims to describe the evidence gathered according to the location where the research was conducted, its design and statistical methods used to analyse the COVID-19 impact. Therefore, the sample of COVID-19-related records indexed in the PubMed database until November 2020, which concomitantly matched the “data” search term, was considered. As a large sample was retrieved (19837 records), automated text analysis and text mining methods were considered to summarise the information. Then, titles and abstracts were screened for inclusion, and more than half of the initial sample was excluded. The main reasons to exclude were not using original data, not being related to the assessment of the impact of COVID-19 on the population's health or well-being, or not having complete information on the title and abstract. This work is intended to highlight gaps in the evidence on the COVID-19 impact on the population's health and well-being, leading to further research that could enhance the countries’ preparedness for future pandemics.

